# Supplementary material for: A Pólya urn approach to information filtering in complex networks
Source: Nat Commun. 2019 Feb 14;10:745. doi: 10.1038/s41467-019-08667-3 (PMC6375975; doi:10.1038/s41467-019-08667-3)
Supplement: Supplementary file 1 — Supplementary Info [file 41467_2019_8667_MOESM1_ESM.pdf]

Supplementary Information for
"A Pólya urn approach to information filtering in complex networks"

Riccardo Marcaccioli,[1] Giacomo Livan[1,2*]

[1] *Department of Computer Science, University College London, London WC1E 6EA, United Kingdom*

[2] *Systemic Risk Centre, London School of Economics and Political Sciences, London WC2A 2AE, United Kingdom*

[*] *Corresponding author. E-mail: g.livan@ucl.ac.uk.*

The PDF file includes:

- Supplementary Notes 1 to 10
- Supplementary Figures 1 to 9
- References

Supplementary Note 1 Explicit expression for the Pólya filter's p-value

The sum in Eq. (2) of the main paper can be computed explicitly in order to derive an explicit expression for the p-value assigned by the Pólya filter to a link with weight w attached to a node

with strength s and degree k. This reads:

$$\begin{aligned}\pi_P(w \mid k, s, a) = \; & 1 - \sum_{x=0}^{w-1} \mathbb{P}(x \mid k, s, a) = \\ = \; & \frac{B\left(\frac{k-1}{a} + s - w, w + \frac{1}{a}\right)}{(s+1)B\left(\frac{1}{a}, \frac{k-1}{a}\right) B(s-w+1, w+1)} \times \\ & \times \; {}_3F_2 \left[\begin{matrix} 1, w + \dfrac{1}{a}, -s + w \\ w+1, -\dfrac{k-1}{a} - s + w \end{matrix} ; 1 \right] ,\end{aligned} \tag{1}$$

where B is the Beta function, and ${}_3F_2$ denotes the generalised hypergeometric function.

It should be noted, however, that the above expression is of little practical use from the numerical viewpoint, due to the presence of the generalised hypergeometric function. Indeed, computing the p-values of the Pólya filter through the sum of the probabilities reported in Eq. (2) of the paper is both faster and more accurate, as values of the beta function can be easily computed by any numerical software with high accuracy. Yet, the above expression is useful to gain analytical insight into the Pólya filter. As a matter of fact, we shall use it in Supplementary Note 3 to derive useful approximations and to prove the relationship between the Pólya and disparity filters.

Supplementary Note 2 The Pólya filter for directed weighted networks

Systems where the directionality of interactions cannot be neglected are usually described in terms of directed weighted networks [1,2]. The difference between weighted directed and weighted undirected networks is that the former are described in terms of a symmetric adjacency matrix W such that $w_{ij} = w_{ji}, \; \forall\, i, j$, where the activity of each node can be specified in terms of a single degree $k_i = \sum_j \mathbf{1}(w_{ij})$ or strength $s_i = \sum_j w_{ij}$. The latter are instead formalized in terms of non-symmetric adjacency matrices, which requires to specify the in- and out-degrees ($k_i^{\text{in}} = \sum_j \mathbf{1}(w_{ji})$ and $k_i^{\text{out}} = \sum_j \mathbf{1}(w_{ji})$, respectively), and the in- and out-strengths ($s_i^{\text{in}} = \sum_j w_{ji}$ and $s_i^{\text{out}} = \sum_j w_{ji}$, respectively) for each node.

The Pólya filter can be easily generalised to weighted directed networks. In the undirected case each weight can be associated with two p-values, one for each of the two nodes the link is attached to. In the directed case we can still associate two p-values to each weight by assessing its statistical significance both as an incoming and as an outgoing link. For example, when testing as an outgoing link, Eq. (1) is easily generalized as (we drop all node indices to keep notation light)

$$\pi_P(w \mid k^{\text{out}}, s^{\text{out}}, a) = \frac{B\left(\frac{k^{\text{out}}-1}{a} + s^{\text{out}} - w, w + \frac{1}{a}\right)}{(s^{\text{out}}+1)B\left(\frac{1}{a}, \frac{k^{\text{out}}-1}{a}\right) B(s^{\text{out}} - w + 1, w + 1)} \times \quad (2)$$

$$\times\, {}_3F_2 \left[\begin{matrix} 1, w + \frac{1}{a}, -s^{\text{out}} + w \\ w + 1, -\frac{k^{\text{out}}-1}{a} - s^{\text{out}} + w + 1 \end{matrix} ; 1 \right], \quad (3)$$

with the replacements $k^{\text{out}} \to k^{\text{in}}$, $s^{\text{out}} \to s^{\text{in}}$ for the test as an incoming link. Both p-values can be tested against the same univariate threshold α. A link is retained by the Pólya filter only when at least one of the two p-values is lower than α.

A link is kept only if at least one of the two p-values is lower than α_B. In the case where $k_i^{\text{out}} = 1$, we keep the directed link connecting i and j only if $\pi_P(w_{ij} \mid k_j^{\text{in}}, s_j^{\text{in}}, a) < \alpha_B$, and vice versa in the case $k_j^{\text{in}} = 1$.

Supplementary Note 3 Generalizing the disparity filter

In this section we explicitly show how the disparity filter [3] can be recovered as a special case of the Pólya filter for $a = 1$. We start by rewriting the p-value associated with a weight w attached to a node with degree k and strength s. For the sake of simplicity, we go back to the undirected case of Eq. (1):

$$\pi_P(w \mid k,s,a) = \frac{B\left(\frac{k-1}{a}+s-w, w+\frac{1}{a}\right)}{(s+1)B\left(\frac{1}{a},\frac{k-1}{a}\right)B(s-w+1,w+1)}\, {}_3F_2\left[\begin{matrix}1, w+\dfrac{1}{a}, -s+w \\ & ;1 \\ w+1, -\dfrac{k-1}{a}-s+w\end{matrix}\right]. \tag{4}$$

In the following, we will repeatedly simplify the above expression by making use of the zero-order Stirling approximation for the ratio of two Gamma functions:

$$\frac{\Gamma\left[x+\alpha\right]}{\Gamma\left[x+\beta\right]} = x^{\alpha-\beta}\left(1+\mathcal{O}\left[\frac{1}{x}\right]\right) \approx x^{\alpha-\beta}\,, \tag{5}$$

which holds for $x \to \infty$.

We first take care of the hypergeometric function in Eq. (4). We start by expanding it in terms of ratios of Gamma functions:

$${}_3F_2\left[\begin{matrix}1, w+\dfrac{1}{a}, -s_i+w \\ & ;1 \\ w+1, -\dfrac{k-1}{a}-s+w\end{matrix}\right] = \tag{6}$$

$$= \sum_{n=0}^{\infty}\frac{\Gamma\left[-s+w+n\right]}{\Gamma\left[-s+w\right]}\,\frac{\Gamma\left[-\frac{k-1}{a}-s+w+1\right]}{\Gamma\left[-\frac{k-1}{a}-s+w+1+n\right]}\,\frac{\Gamma\left[w+\frac{1}{a}+n\right]}{\Gamma\left[w+\frac{1}{a}\right]}\,\frac{\Gamma\left[w+1\right]}{\Gamma\left[w+1+n\right]}\,. \tag{7}$$

We can simplify the last two terms in the above expression:

$$\frac{\Gamma\left[w+\frac{1}{a}+n\right]}{\Gamma\left[w+1+n\right]}\,\frac{\Gamma\left[w+1\right]}{\Gamma\left[w+\frac{1}{a}\right]} \approx w^{\frac{1}{a}+n-(1+n)}w^{1-\frac{1}{a}} = 1\,,$$

where we have assumed $w \gg 1/a$. Putting this result back into Eq. (6) gives:

$${}_3F_2\left[\begin{matrix}1, w+1+\dfrac{1}{a}, -s+w+1 \\ & ;1 \\ w+2, -\dfrac{k-1}{a}-s+w+2\end{matrix}\right] \approx {}_2F_1\left[\begin{matrix}-s+w, 1 \\ & ;1 \\ -\dfrac{k-1}{a}-s+w+1\end{matrix}\right]. \tag{8}$$

Eq. (8) can be now further simplified by making use of the the Chu-Vandermonde identity ${}_2F_1(-n,b;c,1) =$

$\frac{(c-b)_n}{(c)_n}$ (where $(\cdot)_n$ denotes the Pochhammer symbol), which gives:

$$ {}_2F_1 \left[\begin{matrix} -s+w, 1 \\ \\ -\dfrac{k-1}{a} - s + w + 1 \end{matrix} ; 1 \right] = \frac{s - w + \frac{k-1}{a}}{(k-1)/a} \ . \tag{9} $$

Putting Eq. (9) back into Eq. (4), and writing the Beta functions in Eq. (4) as ratios of Gamma functions, allows to write Eq. (4) as the product of the three following ingredients:

$$ \begin{aligned} B\left[\frac{k-1}{a} + s - w, w + \frac{1}{a}\right](s - w + \frac{k-1}{a}) &= \frac{\Gamma\left[\frac{k-1}{a} + s - w + 1\right]\Gamma\left[w + \frac{1}{a}\right]}{\Gamma\left[s + \frac{k}{a}\right]} \\ \frac{1}{(s+1)B\left[s-w+1, w+1\right]} &= \frac{\Gamma\left[s+1\right]}{\Gamma\left[s-w+1\right]\Gamma\left[w+1\right]} \\ \frac{1}{\frac{k-1}{a}B\left[\frac{1}{a}, \frac{k-1}{a}\right]} &= \frac{\Gamma\left[\frac{k}{a}\right]}{\Gamma\left[\frac{1}{a}\right]\Gamma\left[\frac{k}{a} - \frac{1}{a} + 1\right]} \ . \end{aligned} \tag{10} $$

By matching Gamma functions in the numerators and denominators of the above ratios, and making use of the Stirling approximation (Eq. (5)), we can then write down the p-value in Eq. (4) as the product of the following quantities:

$$ \begin{aligned} \frac{\Gamma\left[s - w + \frac{k-1}{a} + 1\right]}{\Gamma\left[s-w+1\right]} &\approx (s-w)^{\frac{k-1}{a}} = s^{\frac{k-1}{a}}\left(1 - \frac{w}{s}\right)^{\frac{k-1}{a}} \ , \qquad s - w \gg \frac{k-1}{a} + 1 \\ \frac{\Gamma\left[w + \frac{1}{a}\right]}{\Gamma\left[w+1\right]} &\approx w^{\frac{1}{a}-1} \ , \qquad w \gg \frac{1}{a}, w \gg 1 \\ \frac{\Gamma\left[s+1\right]}{\Gamma\left[s + \frac{k}{a}\right]} &\approx s^{1-\frac{k}{a}} \ , \qquad s \gg \frac{k}{a}, s \gg 1 \\ \frac{\Gamma\left[\frac{k}{a}\right]}{\Gamma\left[\frac{k}{a} - \frac{1}{a} + 1\right]} &\approx \left(\frac{k}{a}\right)^{\frac{1}{a}-1} \ , \qquad k \gg a - 1 \ , \end{aligned} \tag{11} $$

where on each line we have written the approximations we made use of. Finally, we can put together the above expressions, which gives the result reported in Eq. (6) of the main paper:

$$ \pi_P(w \mid k, s, a) \approx \frac{1}{\Gamma\left[\frac{1}{a}\right]}\left(1 - \frac{w}{s}\right)^{\frac{k-1}{a}}\left(\frac{w\,k}{s\,a}\right)^{\frac{1}{a}-1} \ . \tag{12} $$

All the approximations that we are assuming are written in Equation 11. In Supplementary Figure 1 we show a comparison between the p-values obtained from the Pólya filter (Eq. (4)) and the

above expression in the two networks we consider in our main paper. As it can be seen, the overall agreement is rather good, and larger values of a improve the quality of the approximation, as it can be seen from Eq. 11.

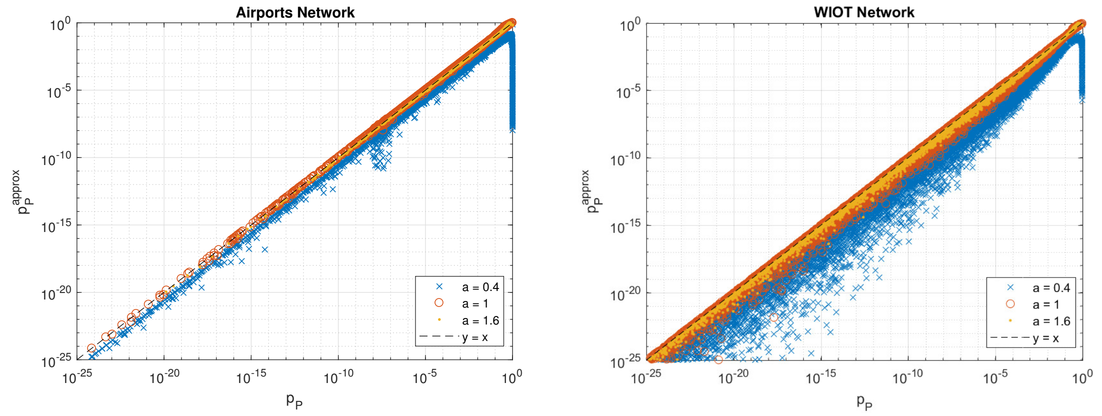

Supplementary Figure 1. Scatter plots of the p-values obtained from the Pólya filter compared with the approximate expression in Eq. (12) for different values of the parameter a.

Supplementary Note 4 Thresholding on r

As discussed in the paper (see Eq. (7) in the Methods Section) there is a soft relationship between the value of the r ratio of a link and the corresponding p-value assigned by the Pólya filter to it. In short, links associated with high values of r tend to be retained, but the opposite does not necessarily hold, i.e., links associated to low values of r can still be validated by the filter and contribute to the overall heterogeneity of Pólya backbones.

In order to highlight this point, in Supplementary Figure 2 we plot the relative difference between the largest connected components of full Pólya backbones, and those of the backbones that would be obtained by thresholding on r. Thresholding is performed by inverting Eq. (7) of

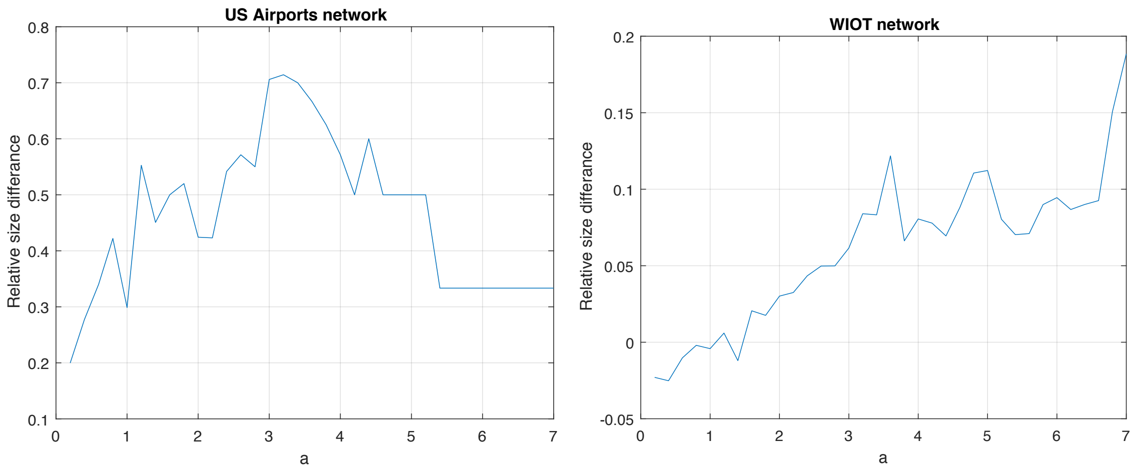

Supplementary Figure 2. Relative difference in the size of the network's largest connected component as measured in the full Pólya backbone and in the backbone obtained by thresholding on r via Eq. (13).

the paper in order to determine the value $r_{\rm thr}$ such that

$$\alpha_B = \frac{e^{-\frac{r_{\rm thr}}{a}} \left(\frac{r_{\rm thr}}{a}\right)^{\frac{1}{a}-1}}{\Gamma\left[\frac{1}{a}\right]} , \tag{13}$$

where α_B is the Bonferroni-corrected multivariate significance level adopted to filter. As it can be seen, both in the case of the US air transport and WIOT networks, thresholding leads to backbones that are considerably more disconnected. This is somewhat to be expected, since thresholding implies producing sparser backbones by discarding links with $r < r_{\rm thr}$ that might be instead validated by the full Pólya filter. Yet, as is particularly apparent in the US air transport network, the sparsification of the largest connected component can be very significant.

The main reason behind this lies in the fact that links associated with high values of r are typically those with a large weight w or those attached to a hub (i.e., with a high k). As such, these links can be easily expected to be validated, unless the parameter a is increased to the point where the network's own heterogeneity is used as null hypothesis (see, for example, the case study on US

air transport network, where all links connecting major hubs are filtered out when setting $a = a_{\mathrm{ML}}$). Conversely, links with lower values of r that are still validated by the Pólya filter correspond to statistically significant *combinations* of w, k, and s, which contribute to the heterogeneity of Pólya backbones (see Supplementary Note 6).

Supplementary Note 5 Maximum Likelihood Estimates

As a parametric approach, the Pólya filter lends itself to optimization procedures aimed at identifying the value of the parameter a most suited to the particular network under study. As mentioned in the main paper, maximium-likelihood estimation (MLE) is a natural option to single out the "nullest" model in the Pólya family for the network under consideration.

This can be achieved by solving

$$a_{\mathrm{ML}} = \arg \max_{a \in [0,\infty)} \mathcal{L}(a; \boldsymbol{w}) \ , \tag{14}$$

where $\boldsymbol{w}$ denotes the sequence of weights in the network, and

$$\mathcal{L}(a; \boldsymbol{w}) = \sum_{i,j=1}^{N} \log \mathbb{P}(w_{ij} \mid s_i, k_i) = \sum_{i,j=1}^{N} \log \left[\binom{s_i}{w_{ij}} \frac{B(\frac{1}{a} + w_{ij}, \frac{k_i - 1}{a} + s_i - w_{ij})}{B(\frac{1}{a}, \frac{k_i-1}{a})} \right] \tag{15}$$

is the log-likelihood function associated with the probability of observing the particular weight sequence under a Pólya process with parameter a.

Solving the optimization problem in (14) with the above function boils down to solving numerically the following equation:

$$\begin{aligned} \sum_{i,j=1}^{N} \Bigg[&-(k_i - 1)\psi \left(\frac{k_i + a s_i - a w_{ij} - 1}{a} \right) + k_i \psi \left(\frac{k_i}{a} + s_i \right) + \\ &(k_i - 1)\psi \left(\frac{k_i - 1}{a} \right) - k_i \psi \left(\frac{k_i}{a} \right) - \psi \left(w_{ij} + \frac{1}{a} \right) + \psi \left(\frac{1}{a} \right) \Bigg] = 0 \ , \end{aligned} \tag{16}$$

where $\psi(x) = \Gamma'(x)/\Gamma(x)$ is the Polygamma function of order 0.

In Supplementary Figure 3 we report ML estimates obtained on synthetic networks. The networks employed in the left panel are characterised by a scale-free topology generated using the BA model [1] and a power-law weight distribution with tail exponent τ. The optimal values a_{ML} clearly show that ML estimates respond to the network's heterogeneity, spanning almost three orders of magnitude ranging from values $a_{\mathrm{ML}} \simeq 10$ in the presence of very strong heterogeneity ($\tau = 1.5$) to $a_{\mathrm{ML}} \simeq 10^{-3}$–$10^{-2}$ in the presence of mild heterogeneity. In the right panel of Supplementary Figure 3 we report the ML estimates on Erdős-Rényi random graphs with a uniform weight distribution $U\,[1, \tau]$, with weights rounded to the nearest integer. As it can been, the estimates are much less sensitive to changes with respect to the previous case, with $a_{\mathrm{ML}} \simeq 0.46$–$0.56$, which implies the *de facto* impossibility to discriminate even between substantially different models when no marked heterogeneity is present in their weight distributions.

Supplementary Note 6 Comparing different Pólya backbones

In this Section we provide numerical evidence in support of the discussion in the Methods Section ("Equivalence of different Pólya backbones") of the main paper, where we argued that the backbones produced by the Pólya filter at different values of a can be made approximately equivalent by tuning the filter's statistical significance. Supplementary Figure 4 shows the univariate statistical significance level α_{DF} that has to be set for a Pólya filter with $a = 1$ (which closely approximates the disparity filter, as demonstrated in the paper) to match the backbones generated by Pólya filters with different values of a at a univariate significance level $\alpha_{\mathrm{PF}} = 0.05$.

As it can be seen, regardless of the multiple testing correction applied (i.e., Bonferroni or FDR), the univariate thresholds required to make the backbones equivalent can differ by several orders of magnitudes. This is true, in particular, in correspondence of notable value of a, i.e., for $a = a^*$ (which denotes the value such that the salience-related metrics O_1 defined in Eq. (5) of

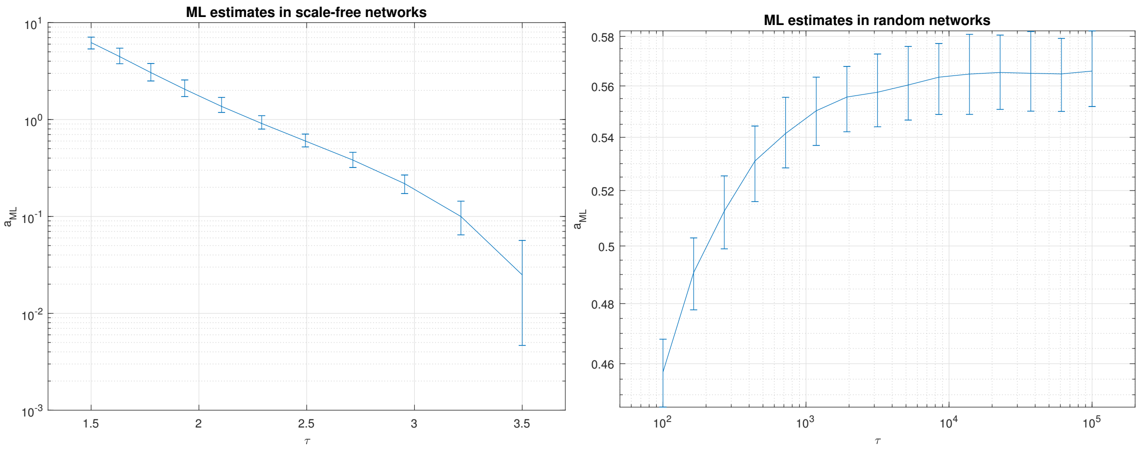

Supplementary Figure 3. ML estimates of the Pólya filter's parameter a. In both cases, the networks are made of $3,000$ nodes and have an average degree of 8. The error bar are 95% confidence intervals obtained through 200 different randomizations of both weights and topology. **Left panel**: ML estimates a^* for Barabasi-Albert networks with a power-law weight distribution with tail exponent τ. **Right panel**: ML estimates a^* for Erdős-Rényi networks with uniform distribution of weights $U\,[1, \tau]$.

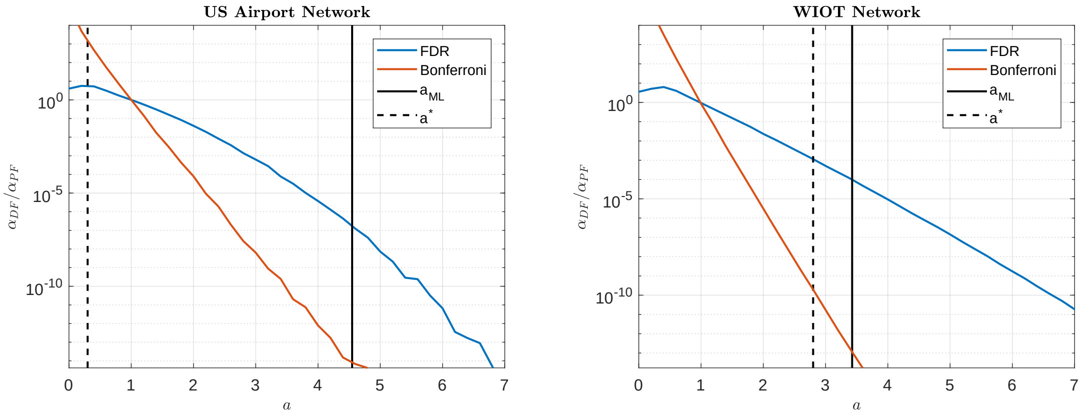

Supplementary Figure 4. Univariate statistical significance level α_{DF} that has to be set for a disparity filter in order to match the backbones generated by Pólya filters with different values of a at a univariate significance level $\alpha_{\mathrm{PF}} = 0.05$. The lines correspond to the ratio $\alpha_{\mathrm{DF}}/\alpha_{\mathrm{PF}}$ when applying the Bonferroni (orange) and false discovery rate (blue) multiple test corrections. The solid vertical line corresponds to the maximum-likelihood value a_{ML} (see Supplementary Note 5), while the dashed vertical line corresponds to the one maximising the salience-related measure O_1 defined in Eq. (5) of the main paper.

the paper is maximised) and for $a = a_{\mathrm{ML}}$ (i.e., when the network's own heterogeneity is used as benchmark for the Pólya null hypothesis).

All in all, these results show that Pólya filters corresponding to different values of a can be made equivalent by tuning their statistical significance. Yet, the above plots show that a difference in a of a few units can lead to dramatic differences in terms of statistical significance (i.e., of ten or orders of magnitude or more). This, in turn, means that the same set of links can have drastically different statistical meanings when generated by different Pólya filters. Indeed, decreasing the univariate threshold α by several orders of magnitude lowers the filter's tolerance to false positives by the same amount, while also causing a much higher false negative. Therefore, a link

discarded by the Pólya filter with parameter a_2 can can still be discarded by the Pólya filter with parameter $a_1 < a_2$ (i.e., a lower tolerance to heterogeneity), but only by making the test extremely conservative.

Supplementary Note 7 Relationship with salience

Link salience is a recently introduced measure of link importance [4], based on the distance between nodes. Given the adjacency matrix W of weighted directed network, where an element w_{ij} represent the strength of the interaction between nodes i and j, the salience is computed through the auxiliary distance matrix D such that $d_{ij} = 1/w_{ij}$ if $w_{ij} > 0$ and 0 otherwise. Once D is known, the salience of a connection (i, j) can be obtained. For a fixed reference node r, the set of weighted shortest paths to all other nodes is called the shortest-path tree matrix $T(r)$, which collects the most effective routes from r to the rest of the network. $T(r)$ is a symmetric $N \times N$ matrix $T(r)$ such that $t_{ij}(r) = 1$ if the link (i, j) is part of *at least* one of the shortest paths starting from r and $t_{ij}(r) = 0$ otherwise. Once all the possible $T(r)\quad r = 1, 2 \ldots N$ matrices have been calculated, the salience of a link (i, j) can be computed as:

$$S_{ij} = \frac{1}{N} \sum_{r=1}^{N} t_{ij}(r) \; . \tag{17}$$

For a large collection of complex networks, it has been found that the distribution of link salience exhibits a peculiar bimodal shape in the unit interval, with most links ending up with $S \approx 0$ or $S \approx 1$. As a result, salience could be used to extract a network backbone, as this would practically not be affected by any particular salience threshold.

Interestingly, the Pólya filter displays an empirical relationship with the salience. In both the WIOT and the US Airport network, we verify that, as we increase the parameter a, the filter has a tendency to retain links with higher salience. We show this in Supplementary Figure 5 by plotting the mean and the skewness of the link salience distribution in both networks computed only in

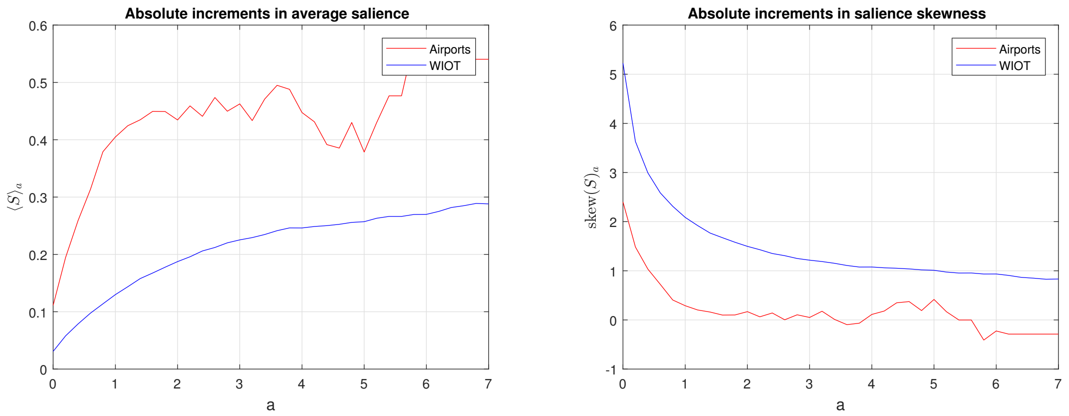

Supplementary Figure 5. **Left panel:** Average salience progressively calculated only in the links included in the backbones: $\langle S \rangle_a = \frac{1}{l_a} \sum_{(i,j)\in \mathcal{P}_a} S_{ij}$ where l_a is the number of links in the backbone $\mathcal{P}_a$. **Right panel:** Skewness of the salience progressively calculated only in the links included in the backbones: $\text{skew}(S)_a = \text{skew}_{(i,j)\in \mathcal{P}_a} S_{ij}$ (S) where l_a is the number of links in the backbone $\mathcal{P}_a$.

the links retained in the Pólya backbones. As it can be seen, the mean increases (not necessary monotonically) while the skewness decreases as a is raised.

The intuition behind this can be found once again in the ratio $r = kw/s$ (Eq. [4] of the main paper). Indeed, we have shown that links associated with a higher r are typically assigned lower p-values by the Pólya filter. The same can be said for the salience, whose scores appear to have a positive and statistically significant rank correlation with the corresponding values of r: corr$(r, s) \approx 0.3$ in the US Airport network, and corr$(r, s) \approx 0.2$ in the WIOT network.

Supplementary Note 8 Additional comparisons between the Pólya filter and other filtering techniques

In this Section we present comparisons between the backbones generated by the Pólya filter and those generated by other filtering techniques on two additional datasets. These are the Florida ecosystem [7] and the High School network [8] (see the Methods Section of the main paper for a description).

As in the main paper, we compare properties of the Pólya backbones obtained at a certain level of statistical significance with those of the backbones obtained (at the same statistical significance) with other methods, i.e., the Hypergeometric Filter (HF) [9], the Maximum-Likelihood filter (MLF) [10], the Enhanced Configuration Model (ECM) based on the canonical ensemble constrained both on degrees and strengths [11], the Noise-Corrected Bayesian filter (NC) proposed in [12], and the Disparity Filter (DF) [3], which in Supplementary Note 3 we have shown to correspond to a large strength approximation of the Pólya filter for $a = 1$. For both the above datasets, we show comparisons across four main dimensions: the fraction of nodes retained in the backbone, the fraction of links retained, the salience-related optimality measure O_1 defined in Eq. (5) of the main paper, and the Jaccard similarity between the B weights retained in the backbone and the top B weights in the original network.

In Supplementary Figure 6 we report the results for the Florida network, while in Supplementary Figure 7 we report results for the High School network. As in the main paper, we see that Pólya backbones are typically sparse, salient, and heterogeneous, and that the other methods we considered do not provide such combination. Indeed, the BF and MLF (whose results are extremely close along all dimensions), tend to preserve exceedingly high fractions of links. This was less evident in the examples shown in the paper (where two other methods ended up validating more links) but is apparent in the examples presented here, where both the BF and MLF validate

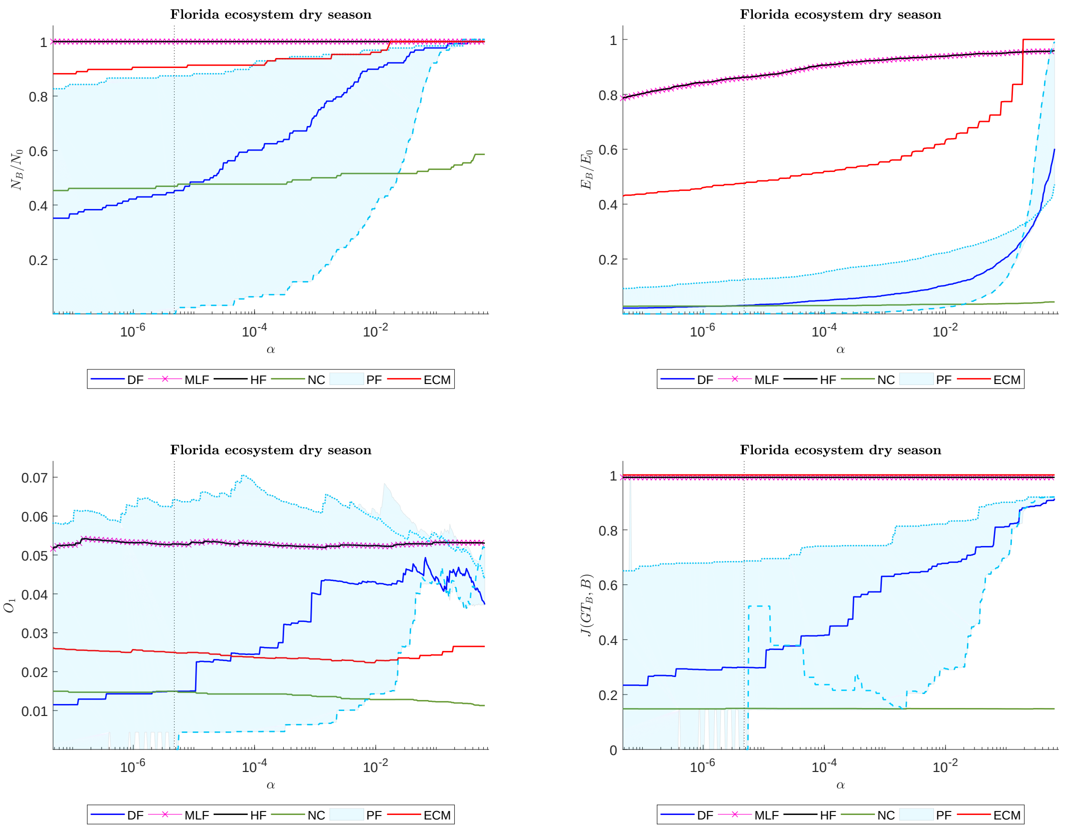

Supplementary Figure 6. Comparisons between the backbones generated for the Florida ecosystem network by the Pólya filter (PF) and other network filtering methods, i.e., the Hypergeometric filter (HF), the Maximum-Likelihood filter (MLF), the Enhanced Configuration Model (ECM), the Noise-Corrected filter (NC), and the Disparity filer (DF). All quantities are shown as a function of the significance level used in the tests. TOP-LEFT: Fraction of nodes retained in the backbones. TOP-RIGHT: Fraction of edges retained in the backbones. BOTTOM-LEFT: Value of the salience-related measure O_1 defined in Eq. (5) of the paper. BOTTOM-RIGHT: Jaccard similarity between the B weights retained in the backbones and the top B weights in the original networks. In all plots the light blue band correspond to all values measured in the Pólya backbone families for $a \in [0.2, 7]$, with the light blue solid (dashed) line corresponding to $a = 0.2$ ($a = 7$).

almost all links in the Florida and HS networks, and do not filter out any node. This, obviously, translates into a very high Jaccard similarity between the weights in the backbone and the top weights in the original network, since almost none of these get filtered out.

The NC method, on the other hand, provides the sparsest backbones of the methods we consider, and such backbones are heterogeneous as testified by the low Jaccard similarity between the weights on the links retained in them and the top links in the original networks. Yet, such links are not salient enough to compensate for such sparsity, as demonstrated by the very low values of the O_1 metrics achieved by the NC method. Hence, such a method provides parsimonious and non-trivial backbones, but it does so at the expense of salience, i.e., filtering out links that are globally important at the network-wide level.

The ECM method represents an intermediate solution between the above. It provides rather parsimonious backbones, but it tends to do so simply by retaining the heaviest links in the network. This is particularly apparent in the case of the Florida network, where the B links retained in the ECM backbone are exactly the heaviest B links in the original network.

The DF corresponds to a large-strength approximation of the Pólya filter for $a = 1$ (see Supplementary Note 3). As such, it obviously occupies an intermediate position in the Pólya family of backbones, and its effectiveness as a filtering tool largely depends on the specific network under study and its heterogeneity. Like other Pólya backbones, it provides more parsimonious representations than other methods. On the other hand, the salience and heterogeneity of DF backbones vary significantly from network to network. For example, in the case of the Florida ecosystem network, the DF yields a heterogenous backbone (as testified by the low value of the Jaccard similarity measure) which, however, is not very salient compared to other Pólya backbones. Conversely, in the case of the High School network, DF backbones are close to being optimal within the Pólya family in terms of salience.

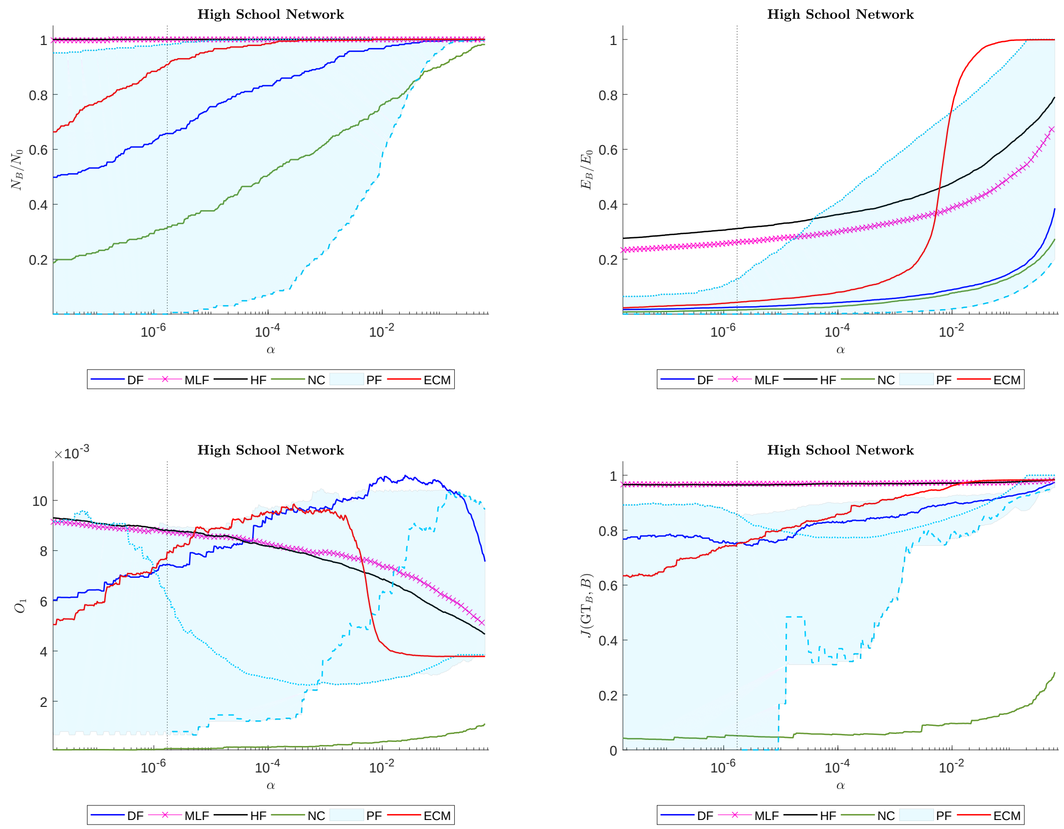

Supplementary Figure 7. Comparisons between the backbones generated for the High School network by the Pólya filter (PF) and other network filtering methods, i.e., the Hypergeometric filter (HF), the Maximum-Likelihood filter (MLF), the Enhanced Configuration Model (ECM), the Noise-Corrected filter (NC), and the Disparity filer (DF). All quantities are shown as a function of the significance level used in the tests. TOP-LEFT: Fraction of nodes retained in the backbones. TOP-RIGHT: Fraction of edges retained in the backbones. BOTTOM-LEFT: Value of the salience-related measure O_1 defined in Eq. (6) of the paper. BOTTOM-RIGHT: Jaccard similarity between the B weights retained in the backbones and the top B weights in the original networks. In all plots the light blue band correspond to all values measured in the Pólya backbone families for $a \in [0.2, 7]$, with the light blue solid (dashed) line corresponding to $a = 0.2$ ($a = 7$).

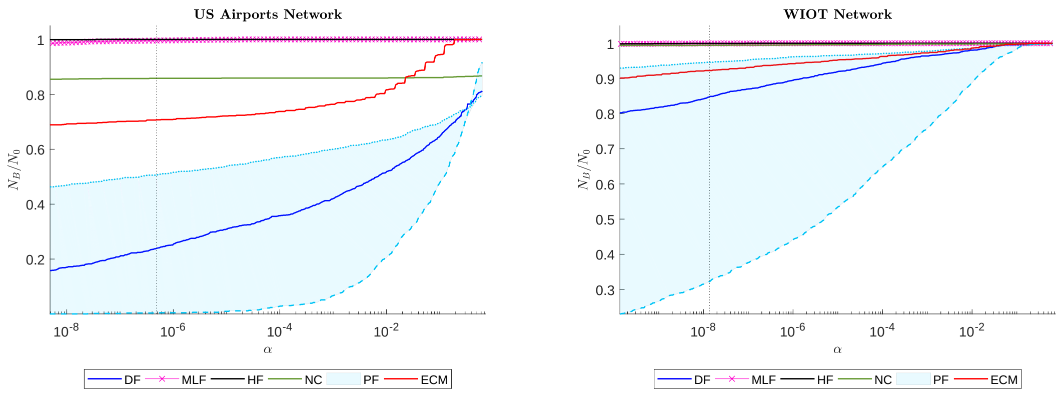

Supplementary Figure 8. Fraction of nodes retained in the Pólya backbones of the US air transport (left) and WIOT (right) networks as a function of the significance level.

All in all, the above results reiterate the message of the main paper, i.e., that the Pólya filter main element of strength is its flexibility. Within reasonable ranges of the parameter a, all Pólya backbones provide a parsimonious representation of the salient relationships in a network, while still retaining weights across multiple scales. Then, depending on the specific application or network, the parameter a can be tuned to generate a backbone which is optimal with respect to a desired criterion.

For the sake of completeness, in Supplementary Figure 8 we report the fraction of nodes retained in the backbones generated by the various methods we considered for the US air transport and WIOT networks, which are not included in the main paper.

Supplementary Note 9 The state-level backbone of the US air transport network

Supplementary Figure 9 shows projections of the US air transport Pólya backbones shown in Fig. 5 of the paper at the state level, i.e. we add a link between two US states (or a self-link on a single state) when there is at least one link connecting two airports located within them. The weight of

the link corresponds to the aggregate weight of all links between the two states. The networks shown correspond to the $a = 1$ (top-left), $a = 2.6$ (top-right), and $a = a_{\mathrm{ML}} = 4.5$ (bottom) Pólya backbones.

As mentioned in the paper, it can be seen that upon increasing a the network becomes increasingly fragmented and disconnected. In particular, as a consequence of the filter's tolerance being tuned to the network's specific heterogeneity, the $a = a_{\mathrm{ML}}$ backbone is essentially made of three main parts: a star-like structure centred around Georgia, a secondary star-like structure centred around North-Eastern states, and a number of smaller disconnected structures mostly involving Western states. In all such structures, the vast majority of relationships are between neighbouring or geographically close states, reflecting the short-haul nature of the $a = a_{\mathrm{ML}}$ backbone. As we mention in the paper, this is due to the fact that, for this value of a, all the relationships which typically characterise the heterogeneity of a complex network have been filtered out. In this case, such relationships correspond to long-haul flights connecting major cities and major hubs.

On the other hand, the retained links form a backbone of regional short-haul connections used by relatively large numbers of passengers. The state of Georgia is the most connected one in the backbone due to the presence of Atlanta's Hartsfield-Jackson airport, which is the busiest in the world and significantly busier than any other US airport. This ensures the "survival" of several links to and from this hub even for $a = a_{\mathrm{ML}}$. Yet, it is notable that, in such a backbone, Hartsfield-Jackson airport only serves as a regional hub for the South-East of the US.

Supplementary Note 10 Predicting trade volume in the WIOT network

Following [5,6], we propose a simple network-based regression model to predict changes in the trade volume between two nodes (representing industrial sectors) in the WIOT network. The model is

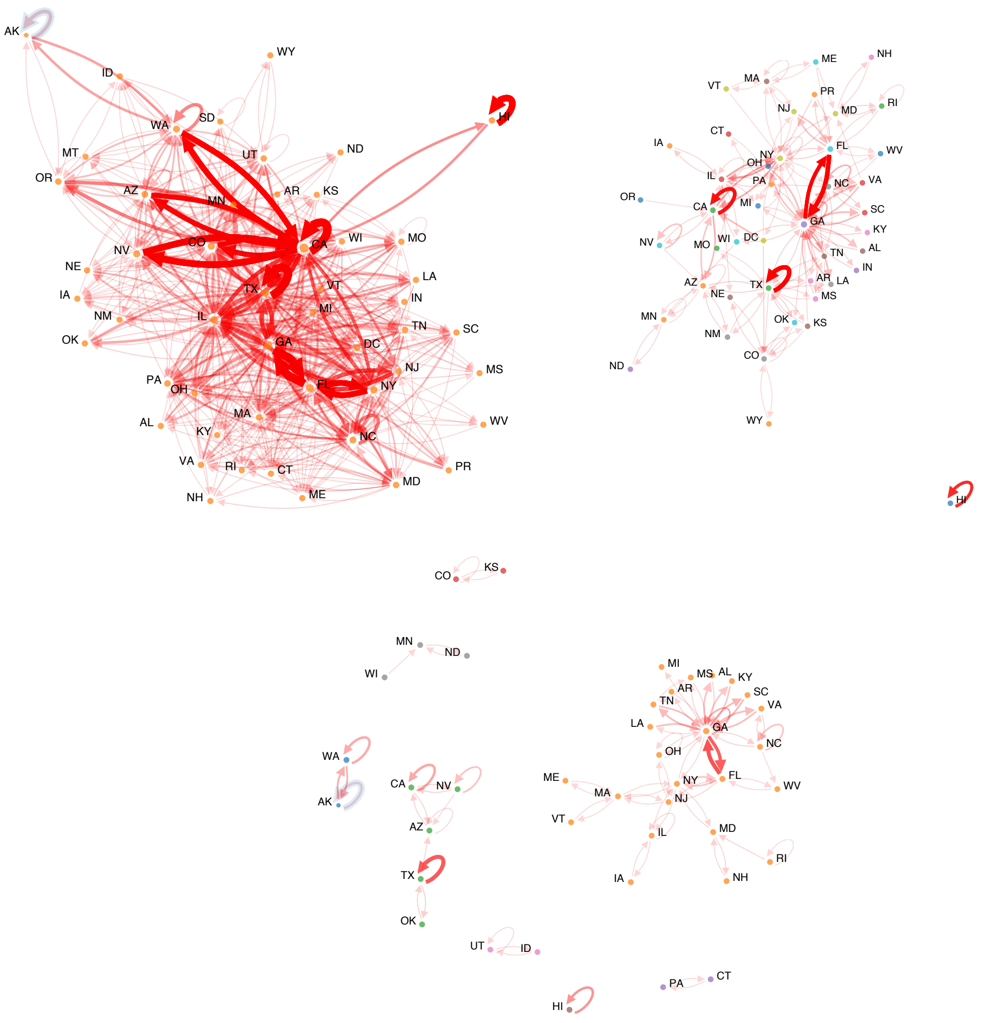

Supplementary Figure 9. Projections of Pólya backbones obtained $a = 1$ (top-left), $a = 2.6$ (top-right), and $a = a_{\mathrm{ML}} = 4.54$ (bottom) at the state level. A link is added between two states when there is at least one link connecting two airports located within them (and similarly for self-link on a single state). Thicker lines correspond to heavier weights, which in turn correspond to the aggregate weight of all links between the two states.

as follows:

$$\log(w_{ij}^{t+\tau}) = \beta_0 + \beta_1 A_{ij}^t + \beta_2 L_{ij}^t \;, \tag{18}$$

where

- w_{ij}^t is the weight on the link between nodes i and j (i.e., the trade volume between the two corresponding industrial sectors) in year t.
- A_{ij}^t is the element of the matrix $A_{ij}^t = w_{ij}^t / \sum_i w_{ij}^t$ in year t, i.e. the trade volume between nodes i and j normalized by the overall outgoing trade volume of node j.
- L_{ij}^t is the year t element of the Leontief matrix, defined as $L = (I - A^T)^{-1}$, where A is defined above and I is the identity matrix. The Leontief matrix is closely related to Katz centrality, and entry L_{ij}^t quantifies the production required from sector j in order to produce one unit of the good produced by sector i.

Note that the regression in Eq. (18) is defined only on links existing at time t (i.e., $W_{ij}^t > 0$). As mentioned in the paper, we calibrated the model over 5 years of data, from 2006 to 2010. We assume time-t values in Eq. (18) to denote the values obtained after such calibration, and in the paper we show the results of the model's prediction for $\tau = 1, 2, 3$ (i.e., for the years from 2011 to 2013).

Supplementary Table 1 shows the results of the model's calibration when performed on the whole WIOT network, and on its Pólya backbones for $a = 1$ and $a = a_{\mathrm{ML}} = 3.4$. As it can be seen, in all three cases the model's coefficients are highly significant, and the model as a whole is able to explain a good portion of the variance in data, as indicated by the R^2 coefficient. Notably, these increase when filtering the network, even though the number N of links used to calibrate the model is reduced by more than two orders of magnitude when going from the full network to the $a = a_{\mathrm{ML}}$ Pólya backbone. Also, upon filtering the network the importance of the weights, encoded in the matrix A_{ij}^t and in its coefficient β_1 in Eq. (18), decreases dramatically. Conversely, the

importance of the Leontief matrix, quantified by its coefficient β_2, increases by roughly a factor 3. This point is particularly significant, since the Leontief matrix is a non-local quantity which assesses the relevance of links from the viewpoint of the whole network they are embedded in. We interpret these results as a sign that Pólya backbones, especially those obtained by tuning the filter to the network's specific heterogeneity, are highly informative, and contain links that are important both locally and globally.

Supplementary Table 1. Regression table of the linear regression model in Eq. (18) calibrated on WIOT network data from 2006 to 2010. The three columns refer to the results obtained when calibrating the model on the full unfiltered network, and on its Pólya backbones for $a = 1$ and $a = a_{\mathrm{ML}} = 3.4$.

	Unfiltered Networks (2006-2010)	Backbones $\mathcal{P}_{a=1}$ (2006-2010)	Backbones $\mathcal{P}_{a=a_{ML}}$ (2006-2010)
β_0	1.61***	6.20***	7.12***
	(0.00096)	(0.0090)	(0.017)
β_1	27.58***	4.52***	3.21***
	(0.043)	(0.064)	(0.079)
β_2	0.018***	0.064***	0.058***
	(0.00011)	(0.00073)	(0.00111)
N	2682840	48853	14784
$R^2 = R^2_{adj}$	0.138	0.196	0.218
F statistic vs constant model	2.16×10^5 ***	5.95×10^3 ***	2.06×10^3 ***

Standard errors in parentheses. Two-tailed test.

*** $p < 0.0001$

Supplementary references

1. Albert, R. & Barabási, A.-L. Statistical mechanics of complex networks. *Reviews of Modern Physics* **74**, 47–97 (2002). 0106096v1.

2. Newman, M. E. J. The structure and function of complex networks. *SIAM Rev* **45**, 167 (2003).

3. Serrano, M. Á., Vespignani, A. & Boguñá, M. Extracting the multiscale backbone of complex weighted networks. *Proceedings of the national academy of sciences* **106**, 6483–6488 (2009). 0904.2389.

4. Grady, D., Thiemann, C. & Brockmann, D. Robust classification of salient links in complex networks. *Nature Communications* **3** (2012). 1110.3864.

5. Carvalho, V. M. & Voigtländer, N. Input diffusion and the evolution of production networks. Tech. Rep., National Bureau of Economic Research (2014).

6. McNerney, J., Savoie, C., Caravelli, F. & Farmer, J. D. How production networks amplify economic growth. *arXiv preprint arXiv:1810.07774* (2018).

7. Ulanowicz, R. E. & DeAngelis, D. L. Network analysis of trophic dynamics in south florida ecosystems. *US Geological Survey Program on the South Florida Ecosystem* **114**, 45 (2005).

8. Mastrandrea, R., Fournet, J. & Barrat, A. Contact patterns in a high school: a comparison between data collected using wearable sensors, contact diaries and friendship surveys. *PloS one* **10**, e0136497 (2015).

9. Tumminello, M., Miccichè, S., Lillo, F., Piilo, J. & Mantegna, R. N. Statistically validated networks in bipartite complex systems. *PLoS ONE* **6** (2011). 1008.1414.

10. Dianati, N. Unwinding the hairball graph: pruning algorithms for weighted complex networks. *Physical Review E* **93**, 012304 (2016).

11. Gemmetto, V., Cardillo, A. & Garlaschelli, D. Irreducible network backbones: unbiased graph filtering via maximum entropy. *arXiv preprint arXiv:1706.00230* (2017).

12. Coscia, M. & Neffke, F. M. Network backboning with noisy data. In *Data Engineering (ICDE), 2017 IEEE 33rd International Conference on*, 425–436 (IEEE, 2017).
